# Feeding Pre-weaned Calves With Waste Milk Containing Antibiotic Residues Is Related to a Higher Incidence of Diarrhea and Alterations in the Fecal Microbiota

**DOI:** 10.3389/fvets.2021.650150

**Published:** 2021-07-08

**Authors:** Martina Penati, Giulia Sala, Filippo Biscarini, Antonio Boccardo, Valerio Bronzo, Bianca Castiglioni, Paola Cremonesi, Paolo Moroni, Davide Pravettoni, Maria Filippa Addis

**Affiliations:** ^1^Dipartimento di Medicina Veterinaria, Università degli Studi di Milano, Lodi, Italy; ^2^Institute of Agricultural Biology and Biotechnology, National Research Council (CNR), Milan, Italy; ^3^Quality Milk Production Services, Animal Health Diagnostic Center, Cornell University, Ithaca, NY, United States

**Keywords:** calf, microbiome, milk, antibiotic residues, gut microbiome, mastitis

## Abstract

The cows receiving antibiotics for intra-mammary infection (IMI) produce milk that cannot be marketed. This is considered waste milk (WM), and a convenient option for farmers is using it as calf food. However, adding to the risk of selecting resistant bacteria, residual antibiotics might interfere with the gut microbiome development and influence gastrointestinal health. We assessed the longitudinal effect of unpasteurized WM containing residual cefalexin on calf intestinal health and fecal microbiota in an 8-week trial. After 3 days of colostrum, six calves received WM and six calves received bulk tank milk (BM) for 2 weeks. For the following 6 weeks, all 12 calves received milk substitute and starter feed. Every week for the first 2 weeks and every 2 weeks for the remaining 6 weeks, we subjected all calves to clinical examination and collected rectal swabs for investigating the fecal microbiota composition. Most WM calves had diarrhea episodes in the first 2 weeks of the trial (5/6 WM and 1/6 BM), and their body weight was significantly lower than that of BM calves. Based on 16S rRNA gene analysis, WM calves had a lower fecal microbiota alpha diversity than that in BM calves, with the lowest *p*-value at Wk4 (*p* < 0.02), 2 weeks after exposure to WM. The fecal microbiota beta diversity of the two calf groups was also significantly different at Wk4 (*p* < 0.05). Numerous significant differences were present in the fecal microbiota taxonomy of WM and BM calves in terms of relative normalized operational taxonomic unit (OTU) levels, affecting five phyla, seven classes, eight orders, 19 families, and 47 genera. At the end of the trial, when 6 weeks had passed since exposure to WM, the phyla Bacteroidetes, Firmicutes, and Saccharibacteria were lower, while Chlamydiae were higher in WM calves. Notably, WM calves showed a decrease in beneficial taxa such as *Faecalibacterium*, with a concomitant increase in potential pathogens such as *Campylobacter, Pseudomonas*, and *Chlamydophila* spp. In conclusion, feeding pre-weaned calves with unpasteurized WM containing antibiotics is related to a higher incidence of neonatal diarrhea and leads to significant changes in the fecal microbiota composition, further discouraging this practice in spite of its short-term economic advantages.

## Introduction

Waste milk (WM) includes low-quality colostrum, transition or post-colostral milk, milk from cows treated for mastitis and other diseases, milk with high somatic cell count (SCC), and other unsalable milk (1). According to European food safety regulations (such as EC Regulation 853 of 2004), this milk is not allowed for direct human consumption or processing into dairy products, with no specific provisions for other uses. Given the clear economic and practical advantages, WM is widely used by farmers as calf food (1, 2). Nevertheless, several countries are issuing guidelines discouraging this practice (i.e., European Commission notice 2015/C 299/04) (1), as the potential presence of anti-microbial residues may increase the risk of maintaining and spreading antimicrobial resistance gene pools in the dairy farm and the environment (3, 4) and expose newborn calves to intestinal diseases (5–7). A further potential issue is the interference of antibiotics and microbial pathogens with the gut microbiome's physiological development in growing calves, with possible consequences on their future health and production performances (5–7).

When antibiotics are administered to adult individuals with a mature gut microbiome, microbial diversity has been shown to decrease significantly, but resilience mechanisms slowly restore the original condition once antibiotics are removed (8). On the other hand, exposure to antimicrobials at an early age may lead to permanent shifts in microbial composition and functions with consequent long-term metabolic alterations (9–12). Therefore, adding to the increased risk of selecting antimicrobial resistance traits, feeding calves with milk containing antimicrobials in the first weeks of life might compromise their intestinal microbiome development impacting gut immunity, gastrointestinal well-being, and ability to metabolize nutrients efficiently (13, 14).

Given its relevance for the dairy industry, previous studies have assessed the impact of WM on calf health and the gut microbiome (3, 13, 14), investigating subtherapeutic levels of antibiotics spiked into milk (14) or milk replacer (13, 15) and pasteurized WM with antibiotic residues at unknown concentrations (3, 16, 17). These studies demonstrated that short-term changes in the microbial taxonomy do occur following WM ingestion, but these are generally limited to disruptions that do not go beyond the genus level (14). However, these studies investigated low or undetermined antibiotic residues and assessed only the time frame of WM feeding.

With these premises, we assessed the impact of WM obtained from cows receiving intra-mammary cefalexin on calf intestinal health and on fecal microbiota diversity and taxonomy during 2 weeks of feeding and after up to 6 weeks after the removal of WM from the diet. To reduce variability, colostrum and WM were standardized and characterized before feeding them to calves. The two-step, 8-week trial included 12 dairy calves enrolled in a commercial farm and managed with standard procedures. For the first 2 weeks, six calves received WM, and six received bulk tank milk (BM); for the following 6 weeks, all calves received the same weaning diet with milk whey and starter feed. Every week for the first 2 weeks and then biweekly for the following 6 weeks, we carried out a complete clinical evaluation and collected fecal swabs for investigating the fecal microbiota composition.

## Materials and Methods

### Farm Description and Ethics Statement

The study was performed on a commercial dairy farm in Northern Italy with a long-standing collaboration with the University of Milan. The farm included 390 lactating Italian Friesian cows. The herd was accredited free from infectious bovine rhinotracheitis (IBR), vaccinated for neonatal diarrhea agents [Rotavec Corona^®^, MSD Animal Health S.r.l., Segrate (MI), Italy], and type-1 and type-2 bovine viral diarrhea virus (BVDV) (Bovela^®^, Boehringer Ingelheim, Milan, Italy). The farm was followed by our University Hospital Clinic and was selected for its very low prevalence of neonatal calf diarrhea (NCD) in the previous 3 months (<1% of cases between newborn calves). The research protocols were reviewed and approved by the Institutional Committee for Animal Care of the University of Milan (protocol number 78_2018). The trial was carried out between March 2019 and June 2019.

### Design of the Feeding Trial and Sample Collection

**Figure 1:**
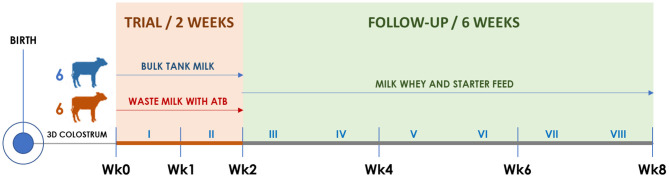
Timeline of the trial illustrating calf groups, diets, and sampling times. Roman numerals indicate weeks. The timing of clinical visits and rectal swab collection is shown according to the trial week, as follows. Wk0: third day of life; Wk1: 10th day of life; Wk2: 17th day of life; Wk4: 31st day of life; Wk6: 45th day of life; Wk8: 59th day of life.

The trial structure is illustrated in Figure 1. Twelve consecutive born male calves were enrolled at birth between March 11 and April 22, 2019. The calves were separated from the dam immediately after birth and received 3 L of the same standardized first colostrum within 6–8 h, followed by 2 L after 8–12 h. During the second and third days of life, calves were fed two times daily with 2.5 L of the same standardized second-day and third-day transition milk (TM), respectively. Colostrum and TM preparation and administration procedures are detailed in section Colostrum, Transition Milk, Waste Milk, and Bulk Tank Milk.

Starting from the fourth day of life, six calves were allocated to the BM group and six to the WM group according to the order of birth. For 2 weeks (Wk0–Wk2; Figure 1), BM calves were fed twice a day with 2 L of fresh unpasteurized BM, while WM calves were fed twice a day with 2 L of an unpasteurized WM lot that was prepared, standardized, and characterized before the beginning of the trial. For the following 6 weeks (Wk2–Wk8; Figure 1), all calves were fed twice a day with 6 L of a commercial milk replacer (Emme Erre Flash 22,5, Tredi Italia S.r.l., Cremona, Italy), and pelleted starter feed (Fly Start, Cortal Extrasoy S.p.A., Cittadella, PD, Italy) was available *ad libitum*. In the first 2 weeks, the calves were housed in individual hutches, while in the last 6 weeks, they were kept in two separated collective pens, one for each experimental group. The WM preparation and administration procedures, as well as the composition of WM, BM, and milk replacer, are detailed in section Colostrum, Transition Milk, Waste Milk, and Bulk Tank Milk.

At birth and on the third day (Wk0), 10th day (Wk1), 17th day (Wk2), 31st day (Wk4), 45th day (Wk6), and 59th day of life (Wk8), all calves were submitted to a complete clinical examination (18) as detailed in section Clinical Examination and Calf Growth Measurements. At each time point, duplicate rectal swabs were collected, refrigerated, brought to the laboratory within 12 h, and stored at −20°C until DNA extraction.

### Colostrum, Transition Milk, Waste Milk, and Bulk Tank Milk

To eliminate possible variables related to colostrum or TM, a pooling strategy was applied as follows. Six liters of good-quality colostrum (Brix >22%) were milked from each of 10 different cows and stored in 500-ml bottles, 12 for each cow. The bottles were identified as colostrum, labeled with the cow number, and frozen at −20°C. Then, 6 L of the second and third milking of the same cows (TM) were again collected in 500-ml bottles, 12 for each cow. The bottles were identified as second-day or third-day TM, respectively, labeled with the cow number, and frozen at −20°C. For colostrum administration, the 3-L morning feeding of each calf was prepared by defrosting and pooling six 500-ml aliquots belonging to cows 1–6, while the 2-L afternoon feeding was prepared by defrosting and pooling four 500-ml aliquots belonging to cows 7–10. The aliquots were gently thawed in a water bath at 45°C for 30 min, mixed, and administered at 35–40°C by oroesophageal tubing. The second-day TM and third-day TM were prepared by mixing aliquots 1–5 for the morning dose (2.5 L for each calf) and aliquots 6–10 for the afternoon dose (2.5 L for each calf) of the respective TM. In this way, all calves received the same colostrum and TM before the start of the feeding trial.

WM was obtained from five cows affected by chronic mastitis (A–E), selected based on a previous bacteriological culture result according to the National Mastitis Council (NMC) guidelines (19). Ten microliters of milk was spread on blood agar plates (5% defibrinated sheep blood), incubated at 37°C, and examined after 24 and 48 h. Colonies were identified based on size, Gram stain, morphology, and hemolysis pattern. The SCC was determined using an automated counter (Bentley Somacount 150, Bentley Instruments, Chaska, MN, USA). The milk collected from the five cows had the following characteristics in terms of SCC and isolated bacteria: cow A, SCC 312,000 cells/ml, *Bacillus* spp.; cow B, SCC 901,000 cells/ml, Non-aureus staphylococci (NAS); cow C, SCC 239,000 cells/ml, *Staphylococcus aureus*; cow D, SCC 5,045,000 cells/ml, *Bacillus* spp.; cow E, SCC 454,000 cells/ml, NAS.

The five cows were subjected to the intramammary administration of 210 mg cefalexin monohydrate (Rilexine 200 T lactation, Virbac S.r.l.) in each quarter for four consecutive milkings, and the milk was collected at each following milking time for a total of 336 L. All the milk was maintained in a refrigerated tank for 36 h from the first to the fourth milking, mixed, aliquoted in 2-L aluminum bags (Perfect Udder^®^ bags, Dairy Tech Inc.) and stored at −20°C until needed. This collection, mixing, and aliquoting procedure ensured the generation of a uniform pooled WM. WM bags were gently thawed in a water bath at no more than 45°C for 45 min and fed to calves at a temperature ranging from 35 to 40°C. BM was collected fresh from the commercial milk tank.

WM and BM were subjected to the determination of total fat, protein, and lactose according to the ISO 9622:2013 (IDF 141) methods and tested for the presence of inhibitors by the Delvotest^®^ SP NT (DSM). WM was further evaluated in triplicate by liquid chromatography–high-resolution mass spectrometry (LC-HRMS) for antibiotic residue detection and quantitation as described by Chiesa et al. (20).

The commercial milk replacer contained milk whey, whey proteins, vegetable oils (coconut, palm), hydrolyzed wheat protein, pregelatinized wheat flour, dextrose, butyric acid esters added with vitamins, oligo-elements, and stabilizers of the intestinal flora *Enterococcus faecium DSM 7134* and *Lactobacillus rhamnosus DSM 7133* at 1 × 10^9^ CFU/kg. The powder was reconstituted according to the manufacturer instructions (125 g/L of powder).

### Clinical Examination and Calf Growth Measurements

Clinical examination and calf growth measurements were performed at the six experimental time points (Wk0–Wk8; Figure 1) by an expert bovine practitioner (GS). At 24 h from birth and on the third day of life, the serum total protein concentration (STP) of each calf was measured to assess the correct transfer of passive immunity (21). A blood sample was collected in a 9-ml tube without anticoagulant from the jugular vein. Samples were allowed to clot, centrifuged at 20°C for 10 min at 900 g, and the STP was measured with a handle refractometer. The calf growth rate was estimated using a heart-girth measuring tape pulled snuggly around the thorax, just caudal to the forelimbs. Obtained measurements were then used to estimate body weight (BW) following the equation proposed by Heinrichs et al. (22). Diarrhea was defined when a calf had visibly watery feces (fecal consistency that permitted feces to run through slightly opened fingers). When a diarrhea episode was detected, fecal samples were collected and submitted to routine diagnostic tests at the local animal health institution (Istituto Zooprofilattico Sperimentale della Lombardia e dell'Emilia-Romagna) for the main agents of NCD: rotavirus and coronavirus by real-time PCR and bacteriological agents by culture.

### DNA Extraction and Generation of 16S rDNA Data

Rectal swabs were thawed, and DNA was extracted using a QIAmp DNA Stool kit (Qiagen, Hilden, Germany) according to the manufacturer instructions with a minor modification. The rectal swabs were dissolved in 1 ml Buffer ASL and shaken at 1,000 rpm (Mixing Block MB-102, CaRlibiotech S.r.l. Rome, Italy) continuously until the stool samples were homogenized. DNA quality and quantity were assessed with a NanoDrop ND-1000 spectrophotometer (NanoDrop Technologies, Wilmington, DE, USA), and the isolated DNA was stored at −20°C until use.

Bacterial DNA was amplified by targeting the V3–V4 hypervariable regions of the 16S rRNA gene (23). PCR amplification of each sample was performed in a 25-μl volume. A total of 12.5 μl of KAPA HIFI Master Mix 2× (Kapa Biosystems, Inc., MA, USA) were used. Then, 0.2 μl of each primer (100 μM) was added to 2 μl of genomic DNA (5 ng/μl). Blank controls (no DNA template) were also included. Amplification and library quantification were carried out as described previously (24).

### Bioinformatic Processing

Demultiplexed paired-end reads from 16S rRNA-gene sequencing were first checked for quality using FastQC (25) for an initial assessment. Forward and reverse paired-end reads were joined into single reads using the C++ program SeqPrep (26). After joining, reads were filtered for quality based on (i) maximum three consecutive low-quality base calls (Phred <19) allowed; (ii) fraction of consecutive high-quality base calls (Phred >19) in a read over total read length ≥0.75; (iii) no “N”-labeled bases (missing/uncalled) allowed. Reads that did not match all the above criteria were filtered out. All remaining reads were combined in a single FASTA file for the identification and quantification of operational taxonomic units (OTUs). Reads were aligned against the SILVA closed reference sequence collection release 123, with 97% cluster identity (27, 28) applying the CD-HIT clustering algorithm (29). A predefined taxonomy map of reference sequences to taxonomies was then used for taxonomic identification along the main taxa ranks down to the genus level (domain, phylum, class, order, family, and genus). By counting the abundance of each OTU, the OTU table was created and then grouped at each phylogenetic level. OTUs with total counts lower than 10 in fewer than two samples were filtered out. All the above steps, except the FastQC reads quality check, were performed with the Quantitative Insights into Microbial Ecology (QIIME) open-source bioinformatics pipeline for microbiome analysis (30). More details on the command lines used to process 16S rRNA-gene sequence data can be found in Biscarini et al. (31).

The 16S rRNA-gene sequencing reads were processed with the QIIME pipeline (30) used to estimate most diversity indices. The Abundance-based Coverage Estimator (ACE) index and sample-based rarefaction were estimated using Python (https://github.com/filippob/Rare-OTUs-ACE.git) and R (https://github.com/filippob/sampleBasedRarefaction) scripts. Plots were generated using the ggplot2 R package (32). Additional data handling and statistical analysis were performed with the R environment for statistical computing (33) and with Microsoft Excel.

### Alpha and Beta Diversity Indices

The fecal microbiota diversity was assessed within (alpha diversity) and across (beta diversity) samples. All indices (alpha and beta diversity) were estimated from the complete OTU table (at the OTU level), filtered for OTUs with more than 10 total counts distributed in at least two samples. Besides the number of observed OTUs directly counted from the OTU table, within-sample microbial richness, diversity, and evenness were estimated using the following indices: Chao1 and ACE for richness; Shannon, Simpson, and Fisher alpha for diversity (34–38); Simpson E and Pielou J (Shannon evenness) for evenness (39). The across-sample microbiota diversity was quantified by calculating Bray–Curtis dissimilarities (40). Prior to calculating the Bray–Curtis dissimilarities, OTU counts were normalized for uneven sequencing depth by cumulative sum scaling CSS (41). Among-groups (BM vs. WM) and pairwise Bray–Curtis dissimilarities were evaluated non-parametrically using the permutational analysis of variance (999 permutations) (42). Details on the calculation of the mentioned alpha and beta diversity indices can be found in Supplementary File 1 and in Biscarini et al. (43).

### Statistical Analysis

The differences between feeding groups were evaluated with SPSS 25.0 (IBM). The distribution of continuous variables was analyzed with the Shapiro–Wilk test. Since the distribution was not normal, data were compared with a non-parametric Mann–Whitney *U*-test. Categoric variables were compared with contingency tables and with the Fisher's exact test (2 × 2 tables), calculating the odds ratio. Statistical significance was considered for *p* < 0.05.

For the microbiome analysis, differences between groups (WM, BM) along time points in terms of OTU abundances and alpha diversity indices were evaluated with a linear model of the following form:

(1) $$\begin{array}{c} \text{y}\_\text{ij}=\text{mu}+\text{treatment}\_\text{j}+\text{e}\_\text{ij} \end{array}$$

where y_ij is the abundance (counts) or index value for each taxonomy (OTU) and alpha diversity metric in animal I belonging to treatment group j, treatment_j is either WM or BM, and e_ij are the residuals of the model. From model (1), *p*-values were obtained to identify those OTUs and alpha diversity indices that were significantly different between treatments along the six time points of the experiment/trial. Alpha diversity indices: value = mu + group + e, within time point.

## Results

### Composition of Waste Milk and Bulk Tank Milk

WM had the following gross composition: SCC 450,000 cells/ml; fat 3.7%; protein 3.6%; lactose 4.7%; microbial inhibitors: present. According to HPLC-MS/MS (20), WM had a residual cefalexin concentration of 727 ppb (727 ng/ml). The mean ± SD composition of BM, based on the routine 10-day measurements received by the farm during its use in the trial, was the following: SCC 284,000 ± 38,742.74 cells/ml; fat 4.23% ± 0.06; protein 3.60% ± 0.00; lactose 4.97 ± 0.06; microbial inhibitors: absent.

### Clinical Findings

During the first 2 weeks of the trial, five out of six (83.33%) WM calves and one out of six (16.67%) BM calves had at least one diarrhea episode. Diarrhea occurred without general impairment of clinical conditions (calves stood securely, presented a strong suckle reflex, and dehydration was <3–5%) (44). Diarrheic calves were treated with oral rehydration solution (ORS) containing 4 g sodium chloride, 20 g dextrose, 3 g potassium bicarbonate, and 3 g sodium propionate between milk feedings, as described by Boccardo et al. (44). According to Constable guidelines (45), antibiotic treatment was omitted because clinical conditions were not severe, no bacterial pathogens of NCD were detected by fecal analysis, and all calves presented an adequate transfer of passive immunity [BM group: 60 g/L of STP, 25% interquartile range (IQR) 58.5 g/L, 75% IQR 61.5 g/L; WM group: 64 g/L of STP, 25% IQR 57.5 g/L, 75% IQR 69 g/L]. During the study period, there were no mortality cases.

At Wk0, the calves enrolled in the BM and WM groups had estimated median weights of 45.41 (25% IQR 43.27; 75% IQR 47.32) and 41.94 (25% IQR 40.61; 75% IQR 48.04), respectively. The difference in weight between the two calf groups at the beginning of the trial was not statistically significant (*p* = 0.29). At Wk1, the difference in estimated weight was significant (p < 0.05) and remained so until the end of the trial (Wk8), when the BM and WM groups had estimated median weights of 85.24 (25% IQR 78.50; 75% IQR 86.50) and 69.99 (25% IQR 62.69; 75% IQR 76.81), respectively.

### Impact of Waste Milk on Fecal Microbiota Diversity

Sequencing of the V3–V4 regions in the bacterial 16S rRNA-gene produced a total of 7,744,670 reads (joined R1–R2 paired-end reads), with an average of 107,564 reads per sample (12 calves × 6 time points = 72 samples). After quality filtering, 1,438,378 sequences were removed, leaving 6,306,292 sequences for subsequent analyses (81.3% overall average retention rate, maximum 86%, minimum 66.3%). Supplementary Table 1 reports the average retention rate and the number of sequences per treatment and time point: the number of sequences ranged from a minimum of 61,592 (±33,344) in the BM group at Wk1 to a maximum of 139,889 (±94,526) in the BM group at Wk4. The initial number of OTUs identified was 10,835; after filtering out OTUs with <10 counts in at least two samples, 3,264 distinct OTUs remained. Supplementary Figure 1 reports the sequence-based and sample-based rarefaction curves generated from the OTU table before filtering (10,835 OTUs), where the observed number of OTUs detected was plotted, respectively, as a function of the number of reads (up to 75,000) in each sample and of the number of samples. Both curves tend to plateau asymptotically, indicating that sequencing depth and the number of samples were adequate. Deeper sequencing or addition of any other sample would not significantly increase the number of new OTUs discovered.

#### Alpha Diversity

**Figure 2:**
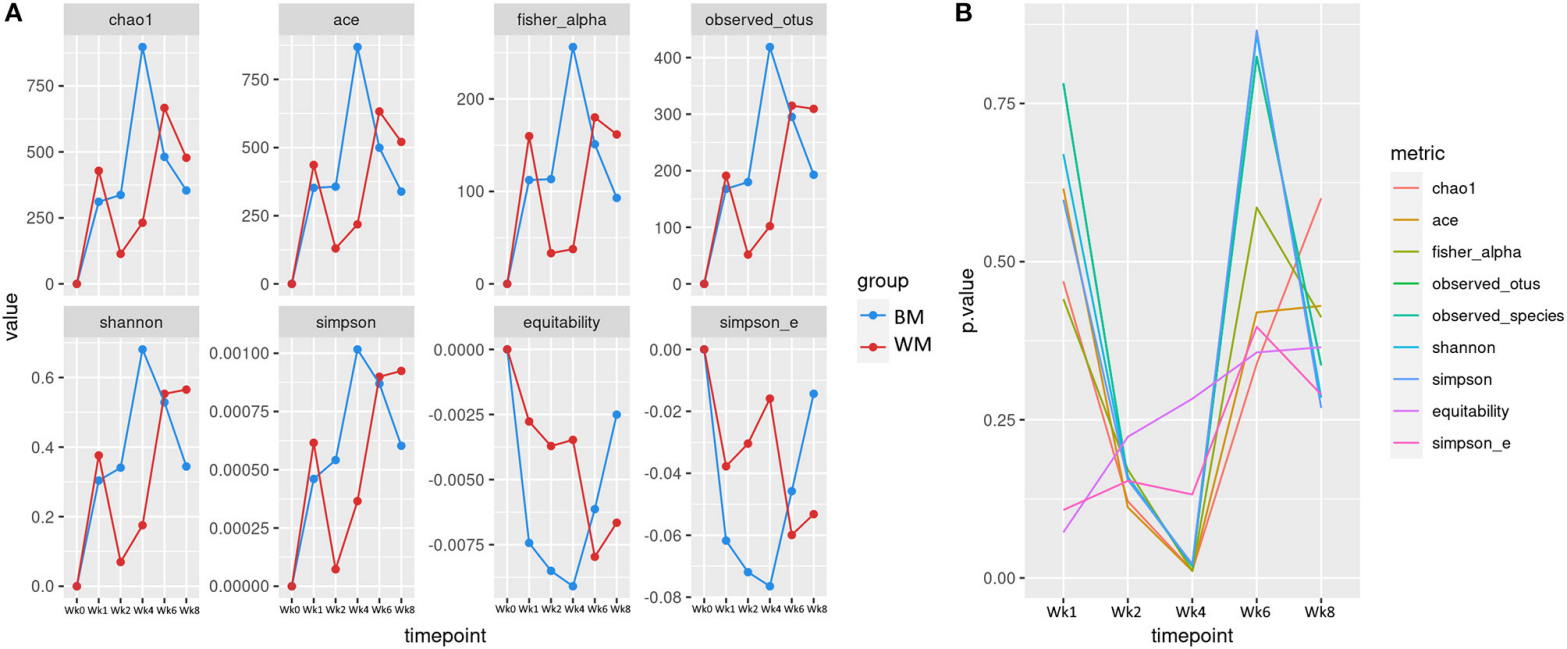
**(A)** Alpha diversity indices at the different trial time points for the two groups: bulk tank milk (BM) and waste milk (WM). Index values are indicated as differences from baseline (Wk0 = 0). **(B)** Statistical significance of alpha diversity indices at the various time points.

Figure 2A illustrates the alpha (within-sample) diversity indices in the fecal microbiota of the two calf groups during the trial, after correcting for baseline. Index values are averages per group, expressed as differences from values at baseline (Wk0). At Wk1, alpha diversity increased in both groups, although slightly less in WM calves. At Wk2, all diversity indices increased in BM and decreased in WM. The difference between groups was further amplified at Wk4, 2 weeks after removing WM from the diet. The two groups reached similar levels at Wk6. At Wk8, the microbiota diversity decreased in both groups, although slightly more in BM. Figure 2B illustrates the significance values for all alpha diversity indices at all the experimental time points. At Wk4, the difference between WM and BM was statistically significant (*p* < 0.05) for all alpha diversity indices, indicating a substantial negative impact on the fecal microbiota diversity that persisted for at least 2 weeks after removing the antibiotic-containing WM from the diet. Equitability and Simpson evenness were significantly different also at Wk1 and Wk2 (*p* < 0.05), respectively.

#### Beta Diversity

**Figure 3:**
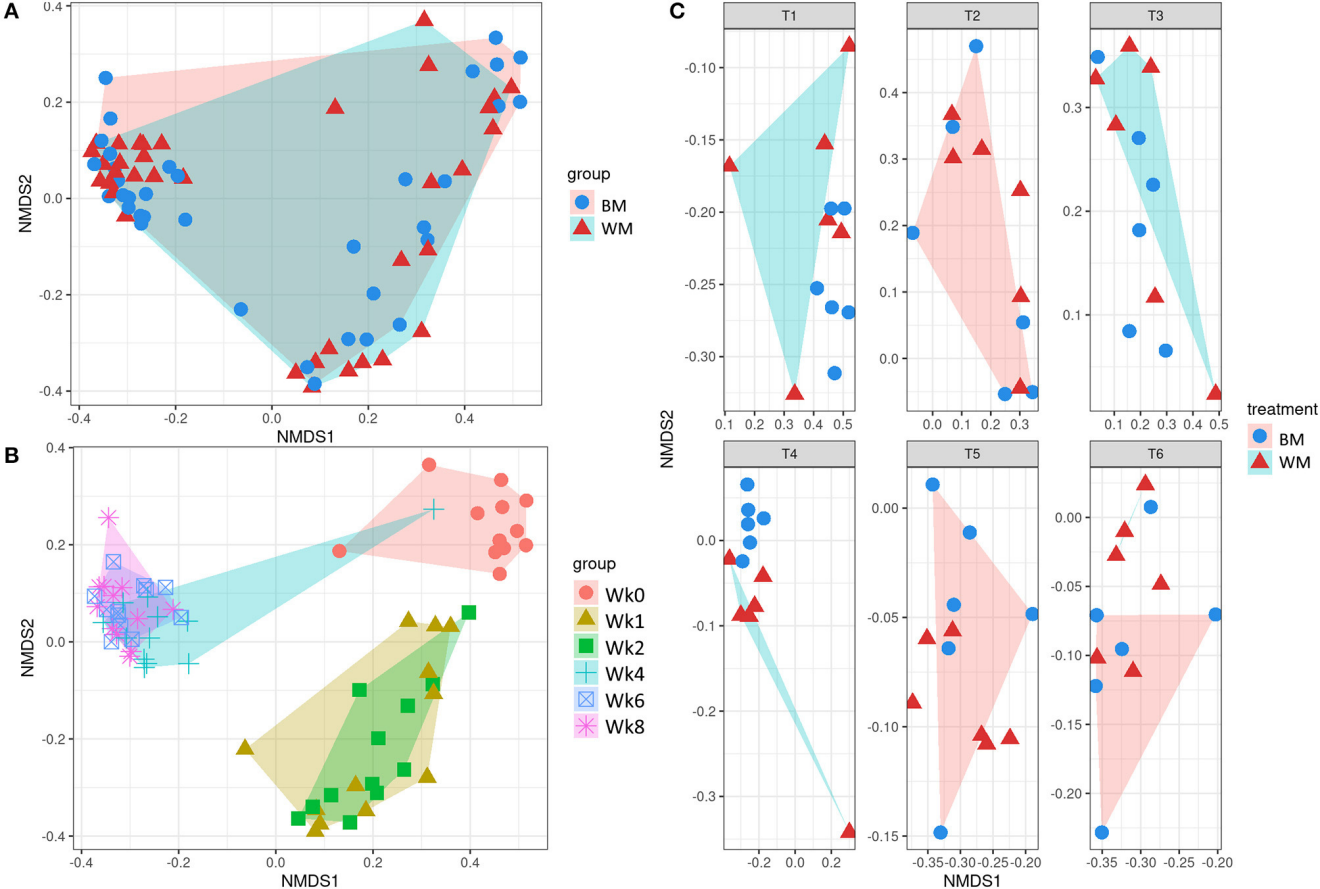
Beta diversity according to treatment **(A)**, time point **(B)**, and treatment-by-time point **(C)**. The legends indicate the color codes and symbols used for the different sample groups [blue circles and pink shading, bulk tank milk (BM); red triangles and turquoise shading, waste milk (WM)] and time points (pink circle, Wk0; brown triangle, Wk1; green square, Wk2; turquoise cross, Wk4; blue box, Wk6; pink asterisk, Wk8).

**Figure 4:**
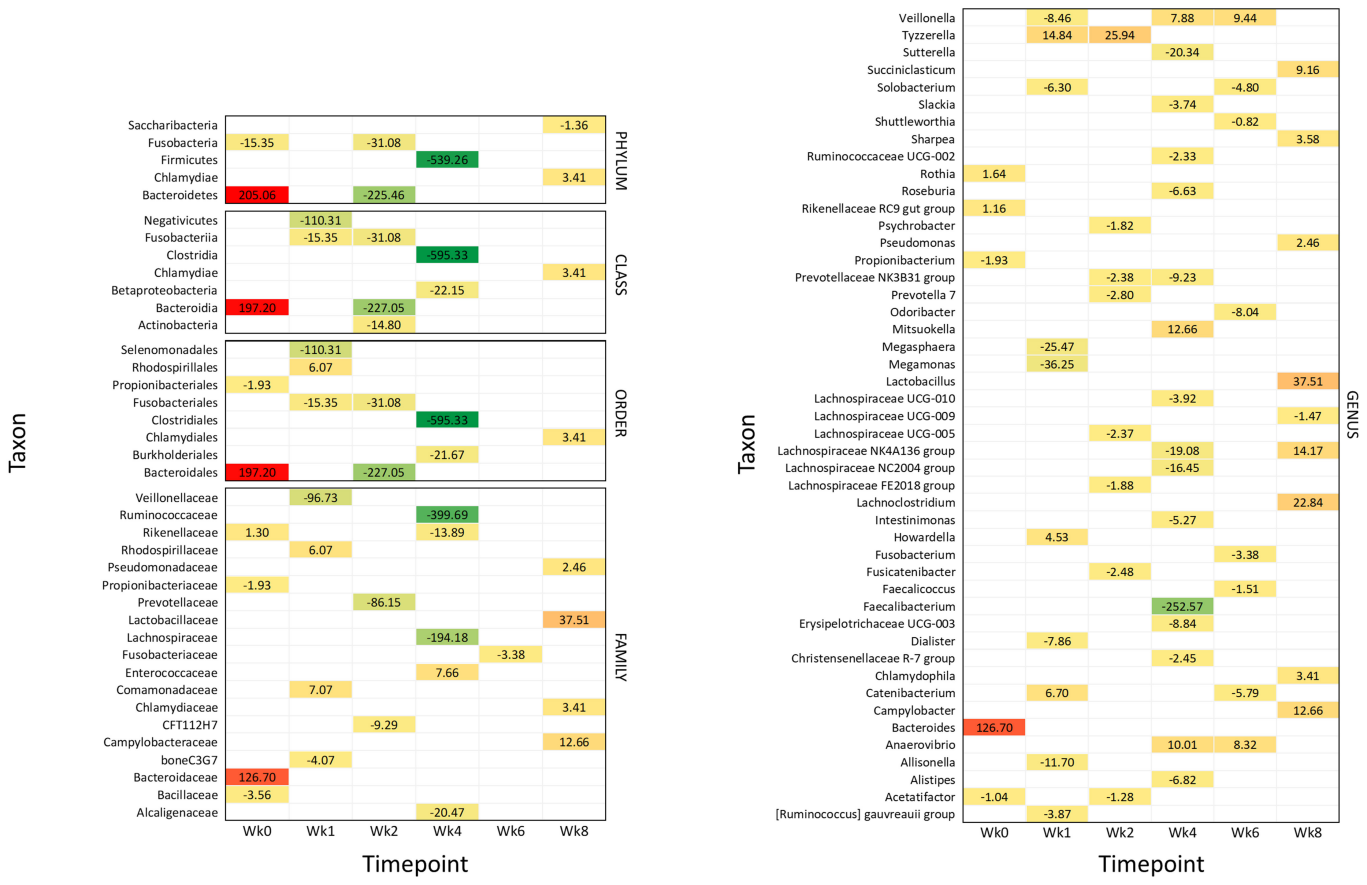
Significantly different taxonomic groups in the fecal microbiota in the two calf groups at the different time points. The results are reported as a heatmap where red indicates a decrease and green indicates an increase of the normalized operational taxonomic unit (OTU) value in waste milk (WM) calves vs. bulk milk (BM) calves for each taxon at the different time points. The normalized OTU values and the statistical significance of the differences are reported in Supplementary Figures 2, 3, respectively.

Figure 3 illustrates the first two dimensions from the (non-metric) multidimensional scaling of the Bray–Curtis dissimilarity matrix, clustering samples by treatment (top left), time point (bottom left), and by treatment-and-time point (right). While the two groups (WM and BM) overlapped extensively, the fecal microbiota evolved by changing significantly during the first 8 weeks of life (*p* = 0.0069505, from PERMANOVA between time points, 999 permutations). Concerning beta diversity between treatments at each time point, the BM and WM groups were separated at Wk4 (Figure 4, right), in line with the alpha diversity results (Figures 2A,B).

### Impact of Waste Milk on the Fecal Microbiota Taxonomy

Figure 4 summarizes all the statistically significant taxonomy changes observed in the fecal microbiota. The changes occurring in WM calves compared to BM calves are illustrated in a heatmap as relative normalized OTU levels for each time point. Normalized OTU levels are detailed in Supplementary Figure 2, while significant values are illustrated in Supplementary Figure 3. As a general observation, and in agreement with the alpha diversity and beta diversity results, most differential taxa were less abundant in WM than in BM calves at all time points, except for the last time point, at Wk8.

#### Wk0 (Age: 3 Days)

At 4 days of life, the phylum Bacteroidetes was significantly more abundant in WM calves; this was reflected in the class Bacteroidia, order Bacteroidales, family Bacteroidaceae, and genus *Bacteroidetes*. The family Rikenellaceae with the related genus *Rikenellaceae* RC9 gut group and *Rothia* were also more abundant. On the other hand, the phylum Fusobacteria and the order Propionibacteriales with the genus *Propionibacterium* were less abundant, together with the family Bacillaceae and the genus *Acetatifactor*.

#### Wk1 (Age: 10 Days)

After 1 week of WM feeding, several taxa showed a significantly lower abundance in WM calves compared to BM calves. These included the two classes Fusobacteria and Negativicutes, the two orders Fusobacteriales and Selenomonadales, the two families boneC3G7 and Veillonellaceae, and the seven genera [*Ruminococcus*] *gauvreauii* group, *Allisonella, Dialister, Megamonas, Megasphaera, Solobacterium*, and *Veillonella*. On the other hand, the order Rhodospirillales and the related family Rhodospirillaceae were more represented, together with Comamonadaceae. The three genera *Catenibacterium, Howardella*, and *Tyzzerella* were also higher.

#### Wk2 (Age: 17 Days)

After 2 weeks of WM feeding, numerous taxa were less abundant in WM vs. BM calves: the two phyla Bacteroidetes and Fusobacteria; the three classes including the related Bacteroidia and Fusobacteriia, together with Actinobacteria; the two related orders Bacteroidales and Fusobacteriales; the two families CFT112H7 and Prevotellaceae; and the seven genera *Acetatifactor, Fusicatenibacter, Lachnospiraceae* FE2018 group and UCG-005, *Prevotella* 7, *Prevotellaceae* NK3B31 group, and *Psychrobacter*. Only the genus *Tyzzerella* was higher in WM vs. BM calves.

#### Wk4 (Age: 31 Days)

The most significant differences between WM and BM calves were observed 2 weeks after the removal of WM from the diet, in line with the alpha diversity and beta diversity results. Numerous taxa were less abundant in WM calves, while only few were more abundant. The most dramatic difference was seen for the phylum Firmicutes and the related class Clostridia, order Clostridiales, family Ruminococcaceae, and genera *Faecalibacterium* and *Ruminococcaceae* UCG-002. Less abundant were also the class Betaproteobacteria with the order Burkholderiales; the three families Alcaligenaceae, Lachnospiraceae, and Rikenellaceae; and the 11 genera *Alistipes, Christensenellaceae* R-7 group, *Erysipelotrichaceae* UCG-003, *Intestinimonas, Lachnospiraceae* NC2004 group, *Lachnospiraceae* NK4A136 group, *Lachnospiraceae* UCG-010, *Prevotellaceae* NK3B31 group, *Roseburia, Slackia*, and *Sutterella*. Only the family Enterococcaceae was higher in WM calves, together with the three genera *Anaerovibrio, Mitsuokella*, and *Veillonella*.

#### Wk6 (Age: 45 Days)

Four weeks after removing WM from the diet, the family Fusobacteriaceae and the six genera *Catenibacterium, Faecalicoccus, Fusobacterium, Odoribacter, Shuttleworthia*, and *Solobacterium* were lower in WM vs. BM calves. On the other hand, the two genera *Anaerovibrio* and *Veillonella* were higher.

#### Wk8 (Age: 59 Days)

Six weeks after removing WM from the diet, the abundance of several taxonomic groups was still different in WM vs. BM calves. In contrast with all the previous time points, however, most differential taxa were significantly higher in WM calves, as follows: the phylum Chlamydiae with the related class Chlamydiae, order Chlamydiales, family Chlamydiaceae, and genus *Chlamydophyla*, the family Campylobacteriaceae with the related genus *Campylobacter*, the family Lactobacillaceae with the related genus *Lactobacillus*, the family Pseudomonadaceae with the related genus *Pseudomonas*, together with the genera *Lachnoclostridium, Lachnospiraceae* NK4A136 group, *Sharpea*, and *Succiniclasticum*. Only the phylum Saccharibacteria and the genus *Lachnospiraceae* UCG-009 were less abundant in WM calves at this time point.

## Discussion

Using WM for feeding calves seems a convenient perspective for the farmer for economic and practical issues, including its disposal, and because of its nutritional qualities. However, as highlighted by numerous researchers and reported in a recent European Food Safety Authority (EFSA) opinion paper, feeding calves with milk containing antibiotic residues presents a significant risk for the development of antimicrobial resistance (1). Another relevant issue is the action on the developing calf gut microbiome, with the potential reduction of overall diversity and the selective inhibition of antibiotic-sensitive microbial groups. Possible consequences are an increased susceptibility to intestinal diseases and the establishment of a dysbiosis with adverse effects on animal health and welfare in later life (1). Gut health results from multiple factors that maintain a disease-free status, and, in this respect, the gut microbiome is crucial (46). Dysbiosis, an imbalance in the gut microbiome, is associated with numerous gastrointestinal and autoimmune diseases (47, 48) and is typically characterized by a reduction in microbial diversity with the loss of beneficial microorganisms and the proliferation of pathobionts (49–51). The general principles governing resilience and dysbiosis seem to apply to most mammals (52–54), but further studies are required to unravel species-specific differences in consideration of the significant differences in the anatomy and physiology of digestion.

### Study Strengths and Limitations

A relevant advantage of this study was the administration of standardized colostrum, TM, and WM, together with WM characterization in terms of antibiotic concentration and nutrient content. In this way, there were no differences in colostrum quality among calves or calf groups, and the composition and antibiotic content of WM remained the same throughout the trial. However, some limitations were also present.

For ethical and practical reasons, the number of calves enrolled in the trial was limited to six per group, and calves were enrolled sequentially, first in the BM and then in the WM group. To offset these issues, the trial was carried out in a reduced time frame, and stringent statistics were applied to highlight the most relevant differences between the groups.

We observed some differences in BM and WM calves' fecal microbiota at the beginning of the trial. Newborn calves have an unstable microbiota, as in the first day of postnatal life, the microbial community's relative composition changes dramatically (55). Therefore, even minimal variations in the hour of sampling in relation to the hour of birth may have led to this result. However, the dramatic changes occurring within 24 h from birth are followed by a relevant increase in the bacterial load, reducing the impact of the time of delivery and reinforcing the reliability of the study findings.

Another point to consider is that WM from cows with mastitis likely had a different milk microbiota in itself than BM. Therefore, the different microbiota in calves fed with WM could have resulted from the microbes being ingested (or the ecological change these microbes created); the study design model used here did not allow us to dissect the effect of drug residues from other factors that differed between WM and BM, such as milk composition and milk microbiota effect on fecal microbiota (56). Furthermore, we cannot rule out a possible influence of the ORS on the WM calves' fecal microbiota (57).

The 16S rRNA gene analysis approach provides information only on bacteria. However, the gut microbiota also includes archaea, protozoa, viruses, algae, and fungi that play crucial roles and participate in maintaining eubiosis (58, 59). For instance, while bacterial communities recover mostly 30 days after heavy perturbations such as an antibacterial treatment, the fungal community may shift from mutualism toward competition (60). Investigations by metagenomics or metaproteomics would also include the non-bacterial components of the calf hindgut microbiome and highlight possible functional profile alterations accompanying the taxonomy changes (61–63). Additionally, results from 16S rRNA-gene sequencing may vary to some extent depending on the software (e.g., QIIME version) and parameters used to process and analyze the data. For instance, the robustness of results to the Phred filtering threshold has been indicated (31), and more comprehensive sensitivity analyses to computer packages and parameters would shed light on these aspects.

Our study was carried out on male calves for animal value issues and ethical aspects due to female calves' more extended life expectancy. Long-term effects in the dairy farm are of interest mainly for what concerns female calves, and therefore gender effects may have to be evaluated more carefully. The breed might also play a role in resilience to intestinal microbiota perturbations (64, 65).

Finally, first-generation cephalosporins are widely used for the intra-mammary treatment of clinical mastitis and are therefore one of the antibiotic classes most likely to be found in WM from cows with bacterial mastitis (66, 67). However, the types and concentrations of antimicrobials in a farm can vary considerably according to management variables and time of the year (13). Some effects observed here might be antimicrobial-dependent, and the presence of other antibiotics in WM, broad-spectrum antibiotics, or the same antibiotics at different concentrations may lead to different results (68). Furthermore, the pasteurization of WM might lead to different results by reducing the microbial load and removing the influence of the WM microbiome. On the other hand, the concentration of antibiotic residues is not changed significantly by pasteurization (69).

### Impact on Calf Diarrhea Incidence and Weight Gain

During the 2 weeks of WM feeding, we observed a significant increase in calf diarrhea incidence. Mitigation of pre-weaned calf mortality is a substantial challenge of the modern cattle industry, and enteric problems are among the major causes of newborn calf death (7, 70). When considering the limitations on prophylactic antimicrobial use (71), it is urgent to minimize the factors that favor the onset of diarrhea and compromise pre-weaned calf gut health, including administration of WM from mastitic cows. A related observation was the negative effect on calf growth. This reduced growth might lead to a slowed start of the animal's productive life (72) and discourages the use of WM also for feeding veal calves. Our results, differ from those of previous reports on this topic. Aust et al. (69) observed that animals fed with WM had a similar growth rate to those fed with milk powder. However, this might be related to the very high incidence of diarrhea observed in our study in the first 2 weeks. The development of juvenile diarrhea is notoriously associated with reduced calf growth (72).

### Alterations in Diversity and Taxonomy of the Microbiota at the Different Time Points

WM feeding led to a dramatic loss in the fecal microbiota's alpha diversity compared to BM. The difference was already evident at Wk2 and highest at Wk4, both concerning richness and uniformity. Therefore, the adverse effects of WM in pre-weaned calves persisted and increased even under a diet with milk replacer containing probiotics integrated with pelleted starter feed, which should instead have led to an increase in the number of bacterial phylotypes in the calf gut (7). Notably, increased microbiome diversity is associated with increased weight gain and a lower incidence of diarrhea in healthy calves at the fourth week of life (73, 74).

Numerous taxa showed significant changes in abundance in calves fed with WM vs. BM, starting from the beginning of the trial and up to 6 weeks after removing WM from the diet. The significant differences observed in the fecal microbiota of WM calves might result from the selective action of cefalexin on some bacterial groups, with a resulting alteration in the microbial equilibria resulting in dysbiosis. On the other hand, the significantly higher incidence of diarrhea in the first weeks of life, due to the elevated antibiotic concentration in WM, could have been responsible for disrupting the microbial ecosystem and the consequent incomplete recovery of the healthy stable state (53).

At Wk1, *Veillonella* was already decreased in WM calves, in agreement with Van Vleck Pereira et al. (14), who observed that *Veillonella* was the only genus significantly decreased in calves fed milk with drug residues at week 1. Their study, however, analyzed WM spiked with low amounts of antibiotics and assessed their effects only during WM feeding. In our study, after 2 and 4 weeks of removing WM from the diet, *Veillonella* increased compared to BM calves. This is undesirable since *Veillonella* produces toxic compounds by fermenting proteins and is negatively associated with short-chain fatty acid (SCFA) production and gut health (75). Also at Wk1, the genus *Tyzzerella* was higher in WM than that in BM calves. Previous studies in humans found a significant increase of *Tyzzerella* and *Tyzzerella 4* in Crohn's disease patients, indicating that this might be a negative occurrence (76). In line with this, another study demonstrated that this genus is overrepresented in patients with an unhealthy diet (77). Other beneficial taxa were decreased, such as *Megamonas* (3), which is also involved in the production of SCFA. SCFAs are crucial for intestinal tissue metabolism and epithelium development and are absorbed into the bloodstream, providing energy for calf metabolism and growth (78).

At Wk2, at the end of the WM feeding period, the Bacteroidetes phylum was significantly less abundant in WM than BM calves. During the pre-weaning period, the rectal microbiota is composed mainly of Firmicutes and Bacteroidetes (79); such a relevant change at this state indicates a strong impact of antibiotics on the microbial equilibria in the calf gut. This agrees with the observations of Maynou et al. (13). In their study, most of the antimicrobials used to treat the cows from which WM originated belonged to the β-lactam family and were mainly cephalosporins. Other studies did not observe disruptions at the phylum level (14). However, this might be due to the higher antibiotic concentration in our WM.

At Wk4, 2 weeks after removing WM from the diet, the phylum Firmicutes was dramatically lower in WM calves than BM calves, and *Faecalibacterium* was the genus with the highest difference in abundance between the groups in the whole study. *Faecalibacterium prausnitzii*, the only known species in this genus, is strongly associated with positive effects on calf health and performance, including the reduction of diarrhea incidence and related mortality rate as well as increased weight gain (80), often together with *Roseburia* that was also less abundant in WM calves (81). These two bacteria are prototypical anti-inflammatory components of the gut microbiota and SCFA producers, especially butyrate, and *Faecalibacterium* represents one of the most abundant bacteria encountered in the feces of healthy animals (82). Calves with a higher abundance of *Faecalibacterium* at a very young age show higher daily weight gain and a lower incidence of diarrhea (74). The whole Firmicutes phylum, mainly concerning the class Clostridia and the order Clostridiales, was dramatically less abundant in WM calves at Wk4. Dysbiosis is characterized by changes entailing a decreased prevalence of Clostridia (obligate anaerobes) (83, 84). Studies in mice showed that a lower relative abundance of Clostridia is associated with intestinal inflammation (54, 85).

At Wk8, when 6 weeks had passed since exposure to the cefalexin-containing WM, alpha diversity was higher for the first time in WM calves than that in BM calves. However, this was accompanied by an increased carriage of taxa associated with veterinary and zoonotic diseases, including *Campylobacter, Chlamydophila*, and *Pseudomonas* (86–89), with relevant consequences on calf health but also in terms of public health, as campylobacteriosis is the most important bacterial food-borne disease in the developed world (90, 91). *Campylobacter* employs many survival strategies and can survive over an extended time in the ruminant gut (91), and its association with *Pseudomonas* may further enhance its survival capabilities (92).

In a general perspective, the increased presence of potential pathogens at the end of the trial, 6 weeks after exposure to the antibiotic-containing WM, may also suggest a status of failing resilience and reduced colonization resistance, that is, the microbiota's competitive exclusion capacities (53, 93). In this respect, the microbiota of WM calves was also more affected by the probiotics contained in the milk substitute, as they showed a significant increase in Enterococcaceae (Wk4, the only increased bacterial taxon above the genus at this time point) and Lactobacillaceae (Wk8, the most intense change observed in terms of increased taxa). In other words, 2 and 6 weeks after receiving WM with antibiotics, the WM calves' gut microbiome was more susceptible to changes due to microorganisms administered with food; that is, the gut microbiome of WM calves was less resilient.

The phylum Saccharibacteria was one of the few taxa decreased in WM vs. BM calves at Wk8. Saccharibacteria, formerly known as TM7 (94), increase in the mature rumen (95), are more abundant in older animals (96), and are part of the core rumen community in lactating dairy cows (97). This further suggests that feeding calves with antibiotic-containing WM may lead to long-term disruptions of the gut microbiota physiology.

## Conclusion

The microbiota plays a crucial role in the development and function of the gastrointestinal tract and gut health (7). It is essential for the proper development of the intestinal epithelium and of the mucus layer (98, 99), the formation of lymphoid structures (100), and the differentiation of immune cells (50, 101). Feeding pre-weaned calves with unpasteurized WM containing residual antibiotics might compromise these processes, impairing gut health and medium-term growth performances. The negative influences observed in the short term on alpha diversity, beta diversity, and taxonomy, together with the longer-term consequences on microbial taxa relevant for ruminal digestive processes and intestinal health, indicate that WM from cows treated with antibiotics should not be given to young calves.

## Data Availability Statement

The data presented in the study are deposited in the EBI European Nucleotide Archive repository, accession number PRJEB42855.

## Ethics Statement

The animal study was reviewed and approved by the Institutional Committee for Animal Care of the University of Milan (protocol number 78_2018). Written informed consent was obtained from the owners for the participation of their animals in this study.

## Author Contributions

MP participated in the feeding trial, data analysis, data interpretation, and manuscript drafting. GS and AB contributed to the feeding trial, clinical monitoring of calves, sample collection, and clinical data analysis and interpretation. PC, BC, and FB contributed to the 16S data generation, analysis, and visualization. VB contributed to the bacteriological culture of milk, selection of cows, and data interpretation. PM and DP contributed to the study conception and design and data interpretation. MA contributed to the study conception, design and coordination, data interpretation and visualization, and manuscript drafting. All authors contributed to the revision and approval of the final manuscript.

## Conflict of Interest

The authors declare that the research was conducted in the absence of any commercial or financial relationships that could be construed as a potential conflict of interest.

## Supplementary Material

Supplementary File 1 Metataxonomic pipeline command lines.

Supplementary Figure 1 Rarefaction curves. The figures show the observed number of detected OTUs plotted as a function of the number of reads in each sample and of the number of samples.

Supplementary Figure 2 Normalized OTU values observed for all taxa showing statistically significant differences in abundance between WM and BM calves. The results are reported as a heatmap where red indicates the highest and green indicates the lowest normalized OTU value observed for each taxon at the different time points.

Supplementary Figure 3 Statistical significance of the differences in normalized OTU abundances between WM and BM calves reported as a heatmap. Intensity of the red color increases with statistical significance.

Supplementary Table 1 Average number of sequences per treatment and time point.
