# Supplementary material for: Feeding Pre-weaned Calves With Waste Milk Containing Antibiotic Residues Is Related to a Higher Incidence of Diarrhea and Alterations in the Fecal Microbiota
Source: Front Vet Sci. 2021 Jul 8;8:650150. doi: 10.3389/fvets.2021.650150 (PMC8298036; doi:10.3389/fvets.2021.650150)
Supplement: Supplementary File 1 — Metataxonomic pipeline command lines. [file Data_Sheet_1.PDF]

APPENDIX 1: METATAXONOMICS PIPELINE COMMAND LINES

To process 16S rRNA gene sequencing data, the following pipeline based on QIIME 1.9 was used [1]. The specific steps and parameters used are detailed below.

Joining paired-end reads

Paired-end reads were joined into single FASTQ files per sample:

```
multiple_join_paired_ends.py --input_dir=<sample_path> --output_dir=./ --include_input_dir_path --
parameter_fp=$PWD/qiime_parameters --read1_indicator _R1 --read2_indicator _R2
```

The method "SeqPrep" for the joining of paired-end reads (https://github.com/jstjohn/SeqPrep) was selected via the parameter file (qiime_parameters):

```
join_paired_ends:pe_join_method  SeqPrep
```

Quality filtering

Joined reads were then filtered for quality and saved into a unique FASTA file for all samples:

```
multiple_split_libraries_fastq.py --demultiplexing_method sampleid_by_file
--input_dir=<multiple_join_paired_ends/> --output_dir=./
--include_input_dir_path --remove_filepath_in_name
--parameter_fp=$PWD/qiime_parameters
```

Quality filter parameter were specified via the parameter file (qiime_parameters):

```
split_libraries_fastq:max_bad_run_length     3     >>     ./qiime_parameters
split_libraries_fastq:min_per_read_length_fraction          0.75          >>
./qiime_parameters      split_libraries_fastq:sequence_max_n      0       >>
./qiime_parameters   split_libraries_fastq:phred_quality_threshold   19   >>
./qiime_parameters
```

OTU picking

OTUs were determined by aligning quality-filtered reads against the QIIME-compatible SILVA reference FASTA file, release 123, with minimum 97% clustering (https://www.arb-silva.de/download/archive/qiime/):

```
pick_closed_reference_otus.py
--reference_fp SILVA123_QIIME/rep_set/rep_set_all/97/97_otus.fasta
--taxonomy_fp SILVA123_QIIME/taxonomy/taxonomy_all/97/raw_taxonomy.txt
--parallel --jobs_to_start=32 --force
--input_fp=<multiple_split_library/>seqs.fna --output_dir=./
```

Filter OTUs

OTUs were filtered by total count across samples greater than 10 distributed in at least 2 samples:

```
filter_otus_from_otu_table.py -i <closed_otupicking/>otu_table.biom -n 10 -s
2 -o ./otu_table_filtered.biom
```

Normalization of OTU counts

To account for uneven sequencing, OTU counts were normalized by cumulative sum scaling (CSS, [2]): normalize_table.py -i

```
<filter_otus/>otu_table_filtered.biom -a CSS -o CSS_normalized_otu_table.biom
```

Alpha diversity

Alpha diversity indexes were estimated from the filtered and normalized OTU table:

```
alpha_diversity.py -i <normalize_otu/>CSS_normalized_otu_table.biom
-m chao1,ace,fisher_alpha,observed_otus,shannon,simpson,equitability,simpson_e
-o ./alpha.txt -t SILVA123_QIIME/trees/97/97_otus.tre
```

Beta diversity

Beta diversity was estimated from the filtered and normalized OTU table:

```
beta_diversity.py -i <normalize_otu/>CSS_normalized_otu_table.biom -m
bray_curtis -o ./ -t SILVA123_QIIME/trees/97/97_otus.tre
```

Sequence-based rarefaction

To check whether sequencing depth was adequate, sequence-based rarefaction curves were generated from the unfiltered OTU table:

```
alpha_rarefaction.py -i <closed_otupicking/>otu_table.biom -m metadatamapping.csv
-o ./ --force --parameter=$PWD/qiime_parameters --parallel
--jobs_to_start=32 --max_rare_depth 75000 --min_rare_depth 100
```

Where metadatamapping.csv is the metadata file (feed supplementation treatments), and max_rare_depth is the median sequence counts per sample. Additional parameters were specified via the parameter file (qiime_parameters):

```
Alpha_diversity:metrics observed_otus,chao1,shannon,simpson,
goods_coverage,ace,fisher_alpha,equitability,simpson_e >> ./qiime_parameters
make_rarefaction_plots:resolution       800 >> ./qiime_parameters
```

References

[1] Caporaso J, Kuczynski J, Stombaugh J, , others Bittinger, K,Caporaso, J G , Kuczynski, J , Stombaugh, J , Bittinger, K , Bushman, F D , Costello, E K , Fierer, N , Pen˜a, A G , Goodrich, J K , Gordon, J I , Huttley GA. QIIME allows analysis of high-throughput community sequencing data. Nature. 2010;7:335–336. doi:10.1038/nmeth.f.303.

[2] Paulson JN, Stine OC, Bravo HC, Pop M. Differential abundance analysis for microbial marker-gene surveys. Nature methods. 2013;10(12):1200–2. doi:10.1038/nmeth.2658.
