# Supplementary figures and images for: Feeding Pre-weaned Calves With Waste Milk Containing Antibiotic Residues Is Related to a Higher Incidence of Diarrhea and Alterations in the Fecal Microbiota
Source: Front Vet Sci. 2021 Jul 8;8:650150. doi: 10.3389/fvets.2021.650150 (PMC8298036; doi:10.3389/fvets.2021.650150)

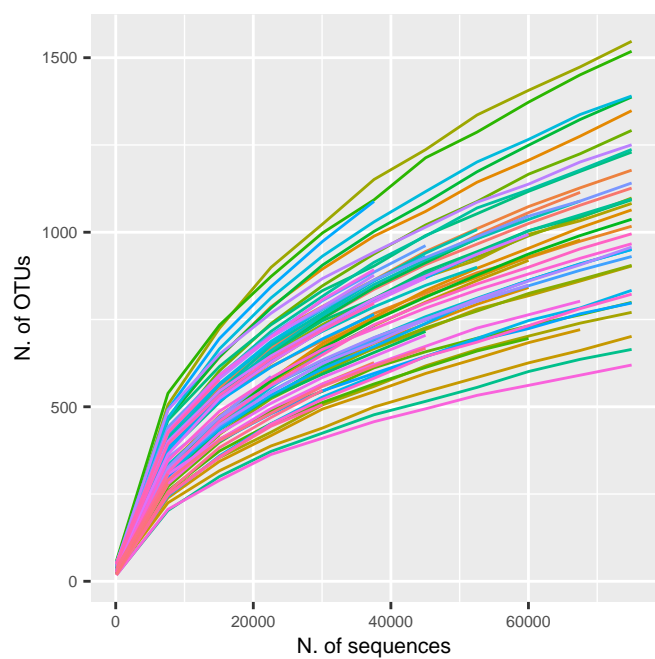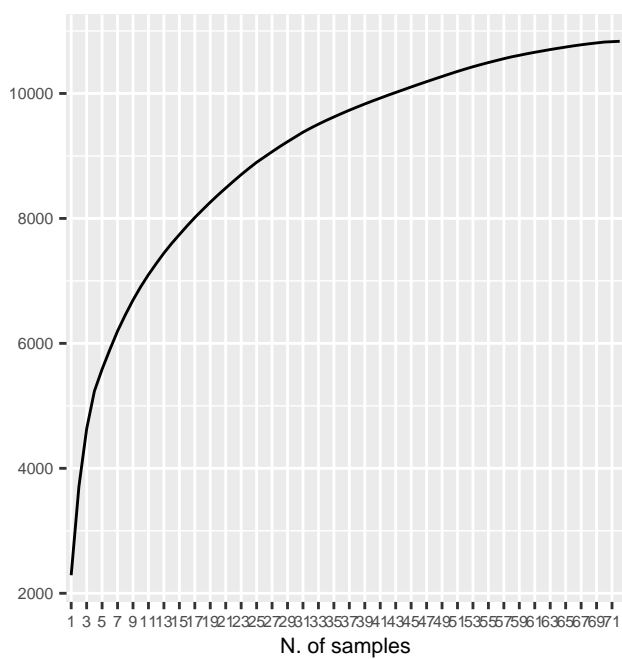

Supplement: Supplementary Figure 1 — Rarefaction curves. The figures show the observed number of detected OTUs plotted as a function of the number of reads in each sample and of the number of samples. [file Image_1.PDF]

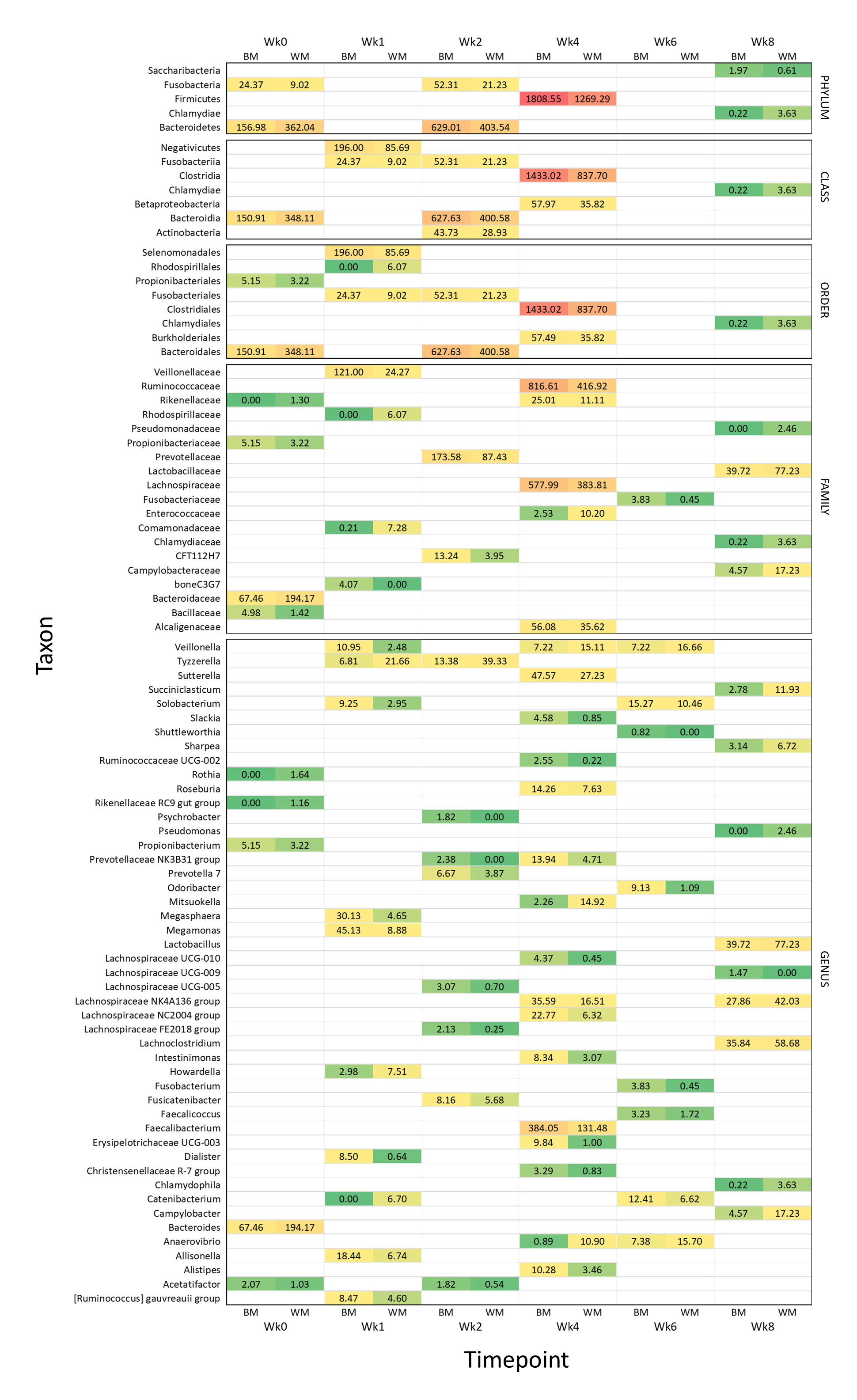

Supplement: Supplementary Figure 2 — Normalized OTU values observed for all taxa showing statistically significant differences in abundance between WM and BM calves. The results are reported as a heatmap where red indicates the highest and green indicates the lowest normalized OTU value observed for each taxon at the different time points. [file Image_2.TIFF]

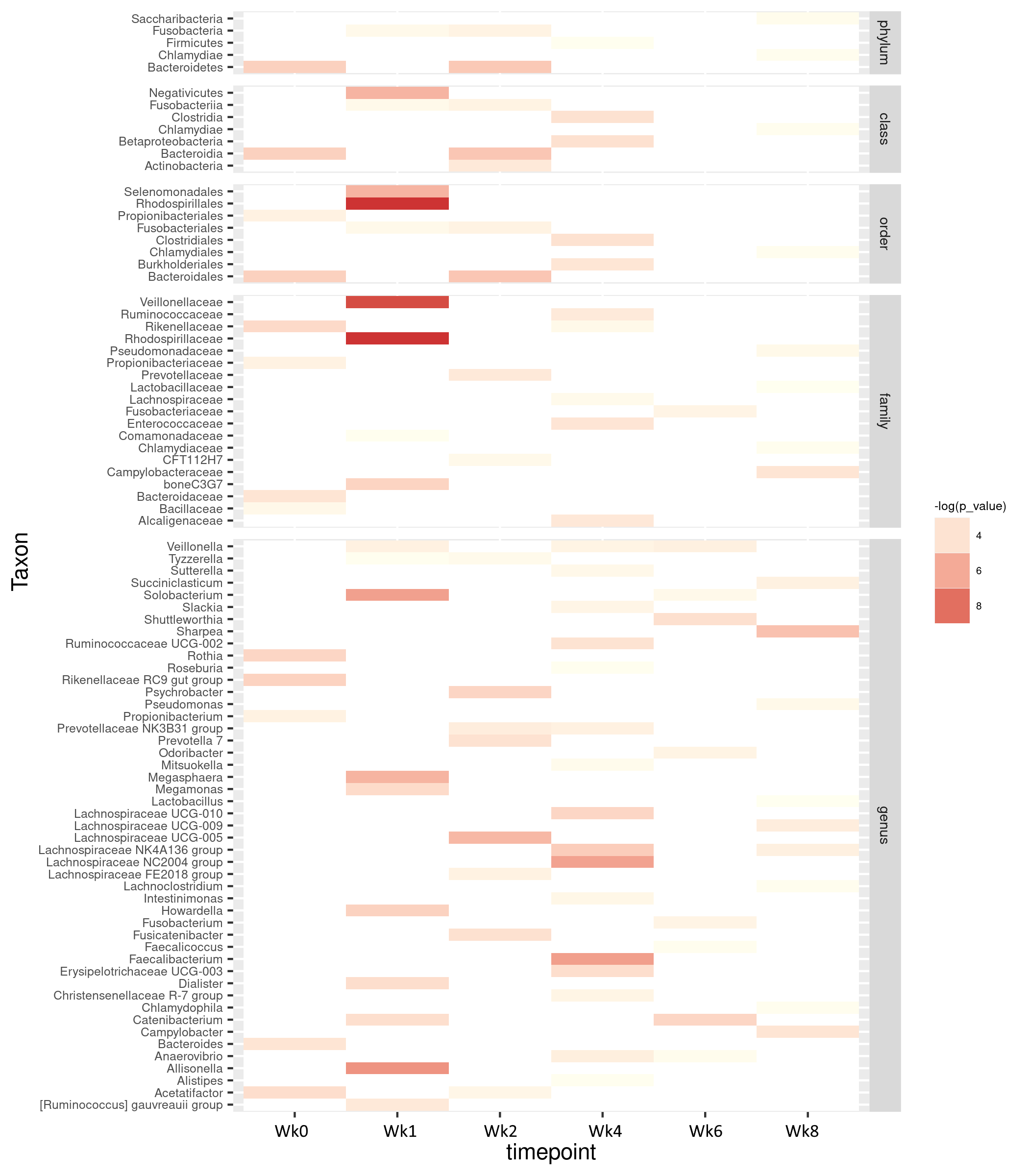

Supplement: Supplementary Figure 3 — Statistical significance of the differences in normalized OTU abundances between WM and BM calves reported as a heatmap. Intensity of the red color increases with statistical significance. [file Image_3.TIFF]
